# Homoeolog expression in polyploid wheat mutants shows limited transcriptional compensation

**DOI:** 10.1111/nph.70646

**Published:** 2025-10-13

**Authors:** Delfi Dorussen, Emilie Knight, James Simmonds, Philippa Borrill

**Affiliations:** ^1^ Department of Crop Genetics John Innes Centre Norwich Research Park Norwich NR4 7UH UK

**Keywords:** active transcriptional compensation, bread wheat (*Triticum aestivum*), durum wheat (*Triticum turgidum* ssp. *durum*), gene duplication, genetic redundancy, polyploidy, transcriptional adaptation

## Disclaimer

The New Phytologist Foundation remains neutral with regard to jurisdictional claims in maps and in any institutional affiliations.

## Introduction

Whole‐genome duplications (WGDs), hybridisations, and small‐scale duplications have resulted in an abundance of gene duplicates in plant genomes – on average, 65% of genes in plant genomes have a paralog (Panchy *et al*., 2016). These duplications can provide opportunities for genetic innovation when mutations cause one of the copies to adopt a novel function (neo‐functionalisation; Birchler & Yang, 2022). Alternatively, the presence of gene duplicates can result in functional redundancy, whereby the effect of a loss‐of‐function mutation in one gene copy is masked by the remaining functional copy. It is debated whether this phenotypic robustness is evolutionarily advantageous or whether selection for lower expression noise results in retention of duplicated genes (Pires & Conant, 2016; Iohannes & Jackson, 2023). Nevertheless, this buffering of deleterious mutations has hindered the functional characterisation of genes (e.g. through gene knockouts) and reduced the variation available for crop breeding (Uauy *et al*., 2017).

Hybridisation and polyploidisation events are widespread in the evolutionary history of the angiosperms and have given rise to major cereal crops such as bread wheat (*Triticum aestivum*) and pasta wheat (*Triticum turgidum* ssp. *durum*; Matsuoka, 2011). Bread wheat is a hexaploid, formed by the merger of three diploid progenitor species. The hexaploid wheat genome therefore consists of three subgenomes (A, B, and D; El Baidouri *et al*., 2017; IWGSC *et al*., 2018). Pasta wheat is a tetraploid, and its genome consists of the A and B subgenomes (El Baidouri *et al*., 2017). As the wheat progenitor species were closely related, many genes are present in highly similar copies across the subgenomes – these duplicate genes formed by polyploidisation are known as homoeologs and share an average nucleotide sequence identity of 97.2% (Schreiber *et al*., 2012; IWGSC *et al*., 2018). Similar to paralogs, functional redundancy can exist between homoeologs, and loss‐of‐function mutations in multiple homoeologs may be required before a phenotype is observed. For example, in hexaploid wheat, loss‐of‐function mutations in all three homoeologs of the *Ms26* gene are required to confer male sterility, while single mutants have no reduction in fertility (Singh *et al*., 2017). Similarly, only triple mutants in *Qsd1* have extended seed dormancy (Abe *et al*., 2019). This redundancy may result in hidden variation in single homoeologs that cannot be observed until mutations in multiple homoeologs are combined (Borrill *et al*., 2015).

However, it remains unclear how phenotypic compensation between gene duplicates occurs. Active transcriptional compensation, the transcriptional upregulation of genes with a high degree of sequence similarity (such as paralogs or homoeologs) in response to a loss‐of‐function mutation in a gene, has been proposed as an explanation for functional redundancy (Sztal & Stainier, 2020). Such transcriptional compensation has been observed in multiple species, including *Caenorhabditis elegans* (Serobyan *et al*., 2020) and *Danio rerio* (zebrafish; El‐Brolosy *et al*., 2019; Ma *et al*., 2019). The degree of transcriptional compensation between paralogs is also hypothesised to underlie the penetrance of mutations – for example, in zebrafish, a greater degree of craniofacial distortion was observed due to mutation of *MEF2CA* in the absence of upregulation of the *MEF2C* paralogs (Bailon‐Zambrano *et al*., 2022).

Transcriptional compensation between paralogs has also been documented in numerous plant species, for example in the *CLE* gene family. Transcriptional upregulation of *CLE9* (a *CLV3* paralog) is observed in *clv3* mutants in *Solanum lycopersicum* (tomato), *Petunia hybrida* (petunia), and *Physalis grisea* (groundcherry; Rodriguez‐Leal *et al*., 2019; Kwon *et al*., 2022). As such, *clv3* mutants in these species have weaker phenotypes than the *clv3* mutant in *Nicotiana benthamiana* (tobacco), in which *CLE9* has become pseudogenised (Kwon *et al*., 2022). However, active compensation is not always observed between paralogs in plants – for example, in *Arabidopsis thaliana*, there was no transcriptional upregulation of *RPL23aA* in response to the knock‐out of its paralog *RPL23aB*, or vice versa (Degenhardt & Bonham‐Smith, 2008; W. Xiong *et al*., 2020). It is unknown whether active transcriptional compensation occurs in wheat or other polyploid plants, buffering the effects of mutations in individual homoeologs.

Here, we assessed whether transcriptional compensation is prevalent between homoeologs in hexaploid and tetraploid wheat using ethyl methanesulfonate (EMS) mutagenised Targeting Induced Local Lesions in Genomes (TILLING) lines (Krasileva *et al*., 2017). Each wheat TILLING line has a large number of mutations (> 5000 in the hexaploid cultivar Cadenza; Krasileva *et al*., 2017), allowing us to simultaneously screen the effect of many mutations. We performed RNA‐sequencing and differential gene expression analysis to determine whether genes are frequently upregulated in response to a loss‐of‐function mutation in one of their homoeologs. We found no evidence for widespread active transcriptional compensation between homoeologs, with a rate of *c*. 3% in hexaploid and tetraploid wheat, indicating that such a mechanism is unlikely to be the primary cause of functional redundancy between homoeologs in polyploid wheat.

## Results

**Fig. 1:**
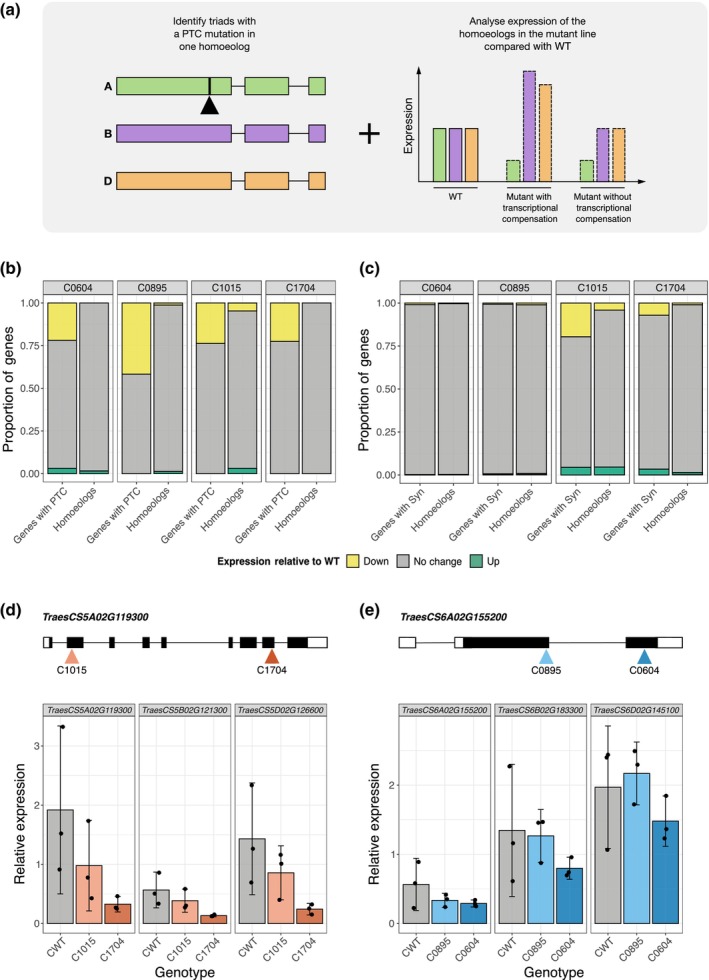
Absence of active transcriptional compensation between homoeologs in response to premature termination codon (PTC) mutations in hexaploid wheat (*Triticum aestivum*). (a) Homoeolog groups with a PTC mutation in one homoeolog were identified in mutagenised wheat lines, and homoeolog‐specific expression was analysed relative to the wild‐type (WT) control. This allowed us to identify homoeolog groups with and without transcriptional compensation. (b) Expression of genes with a PTC mutation (left bar) and their homoeologs (right bar) in each of the mutagenised Cadenza lines. (c) Expression of genes with a synonymous (Syn) mutation (left bar) and their homoeologs (right bar) in each of the mutagenised lines. In (b) and (c), the proportion of genes that are downregulated (false discovery rate (FDR)‐adjusted *P*‐value < 0.05) is shown in yellow, upregulated (FDR‐adjusted *P*‐value < 0.05) in green, and no change in expression in grey, all relative to Cadenza WT. (d) The location of PTC mutations in *TraesCS5A02G119300* in the C1015 and C1704 lines and the relative expression of *TraesCS5A02G119300* and its homoeologs (*TraesCS5B02G121300* and *TraesCS5D02G126600*) in Cadenza WT (CWT; grey), C1015 (light orange), and C1704 (dark orange). (e) The location of PTC mutations in *TraesCS6A02G155200* in the C0895 and C0604 lines and the relative expression of *TraesCS6A02G155200* and its homoeologs (*TraesCS6B02G183300* and *TraesCS6D02G145100*) in Cadenza WT (CWT; grey), C0895 (light blue), and C0604 (dark blue). In (d) and (e) in the gene schematics, black rectangles represent exons, white rectangles represent untranslated regions, and lines represent introns. Error bars represent the 95% confidence interval around the mean, estimated as 1.96 × SE.

To test whether active transcriptional compensation occurs between homoeologs in hexaploid wheat, we screened four representative EMS‐mutagenised TILLING lines (cv Cadenza) to identify homoeolog groups with a premature termination codon (PTC) mutation in one of the homoeologs. PTC mutations are expected to cause truncation of the protein encoded by the gene, thus resulting in loss of function that could be associated with upregulation of the gene's homoeologs to provide a buffering effect. Each line underwent two generations of single seed descent (SSD) to increase homozygosity. Differential expression of the gene affected by the PTC mutation and its homoeologs in the SSD TILLING line relative to wild‐type (WT) was determined by analysis of RNA‐sequencing data (Fig. 1a).

Across the four mutagenised SSD lines (C0604, C0895, C1015, and C1704), we identified 158 unique homoeolog groups with a PTC mutation in one homoeolog. In 20.6% (C0604) to 41.7% (C0895) of cases, the homoeolog with the PTC mutation was downregulated in the mutagenised line relative to WT (false discovery rate (FDR) adjusted *P*‐value < 0.05; Fig. 1b). However, despite our relaxed threshold for detection, we did not observe widespread upregulation of the homoeologous genes – across the mutagenised lines, we identified only four homoeolog groups (2.5%) in which at least one of the homoeologs was upregulated (FDR‐adjusted *P*‐value < 0.05; Fig. 1b; Supporting Information Table S1). The PTC mutations associated with homoeologous upregulation affected groups with various homoeolog expression patterns in WT, suggesting that this effect is not associated with the loss of dominantly expressed homoeologs (Table S1).

Furthermore, the proportion of homoeolog groups with upregulated homoeologs was not significantly different for homoeolog groups affected by PTC mutations or synonymous mutations (2.5% of homoeolog groups with a PTC mutation, 3.8% of homoeolog groups with a synonymous mutation; chi‐squared test *P*‐value = 0.401; Fig. 1c). Active transcriptional compensation is not expected to occur in response to synonymous mutations as they are not expected to affect protein function. This suggests that active transcriptional compensation is not the default mechanism for buffering single homoeolog mutations.

We found a similar lack of active transcriptional compensation in an independent RNA‐sequencing dataset of two EMS‐mutagenised lines produced by H. C. Xiong *et al*. (2020). Thirty homoeolog groups with a PTC mutation were identified, of which only one (3.3%) showed upregulation of the homoeologs (Fig. S1a; Table S1). Again, there was no significant difference between the proportion of upregulated homoeologs in groups affected by a PTC mutation compared with those affected by a synonymous mutation (3.5% of homoeolog groups with a synonymous mutation, chi squared test *P*‐value = 0.951; Fig. S1b).

Next, we investigated whether the location of the PTC mutation within the transcript affects whether active transcriptional compensation occurs. We hypothesised that PTC mutations occurring earlier within the coding sequence would have a greater impact on protein function and therefore promote more compensatory upregulation. We identified genes with multiple PTC mutations across the four Cadenza mutagenised lines, differing in their position within the gene. Accordingly, we found *TraesCS5A02G119300* (with an early PTC in C1015 and a late PTC in C1704) and *TraesCS6A02G155200* (with an early PTC in C0895 and a late PTC in C0604; Fig. 1d,e; Table 1). The relative expression of *TraesCS5A02G119300*, *TraesCS6A02G155200*, and their homoeologs in the mutagenised lines compared with WT was determined by reverse transcription polymerase chain reaction (RT‐qPCR). We found no significant effect of genotype on expression of any of the homoeologs (ANOVA, *P*‐value > 0.05; Fig. 1d,e). This was further confirmed by the RNA‐sequencing results (FDR‐adjusted *P*‐value > 0.05; Fig. S2a,b). Moreover, in those cases where homoeologous upregulation was observed, the PTC mutations were distributed throughout the coding sequence (Table S1). Overall, we found no evidence for widespread active transcriptional compensation between homoeologs in hexaploid wheat.

**Fig. 2:**
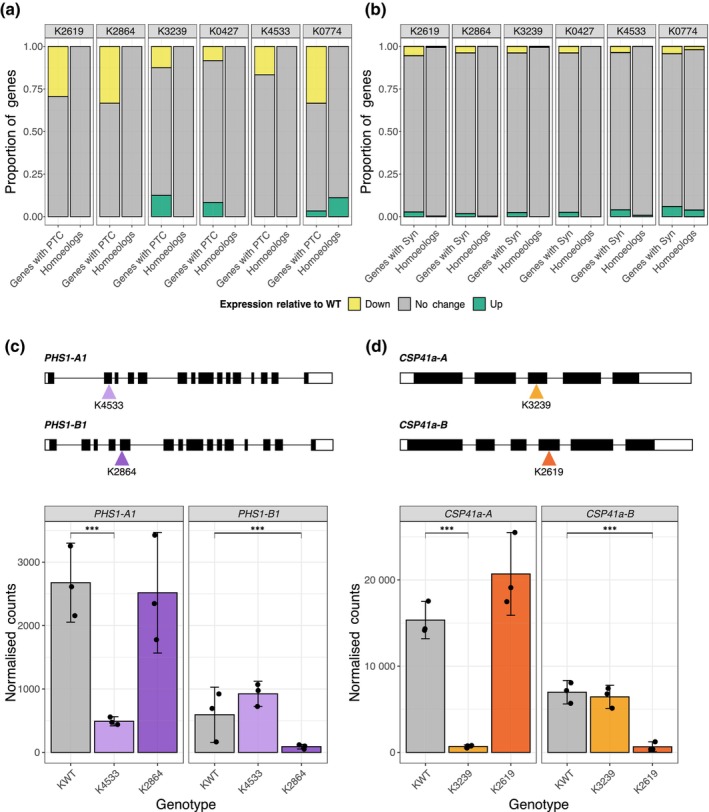
Absence of active transcriptional compensation between homoeologs in response to premature termination codon (PTC) mutations in tetraploid wheat (*Triticum turgidum* ssp. *durum*). (a) Expression of genes with a PTC mutation (left bar) and their homoeologs (right bar) in each of the mutagenised Kronos lines. (b) Expression of genes with a synonymous (Syn) mutation (left bar) and their homoeologs (right bar) in each of the mutagenised lines. In (a) and (b), the proportion of genes that are downregulated regulated (false discovery rate (FDR)‐adjusted *P*‐value < 0.05) is shown in yellow, upregulated (FDR‐adjusted *P*‐value < 0.05) in green, and no change in expression in grey, all relative to Kronos wild‐type (WT). (c) The location of PTC mutations in *PHS1‐A1* and *PHS1‐B1* in K4533 and K2864 respectively and the expression of *PHS1‐A1* and *PHS1‐B1* in Kronos WT (KWT; grey), K4533 (light purple), and K2864 (dark purple). (d) The location of PTC mutations in *CSP41a‐A* and *CSP41a‐B* in K3239 and K2619 lines respectively and the expression of *CSP41a‐A* and *CSP41a‐B* in Kronos WT (KWT; grey), K3239 (light orange), and K2619 (dark orange). In (c) and (d) in the gene schematics, black rectangles represent exons, white rectangles represent untranslated regions, and lines represent introns. Error bars represent the 95% confidence interval around the mean, estimated as 1.96 × SE. Asterisks show significant differences in gene expression between gene expression; ***, *P* < 0.001 (DESeq2 *P*‐value with FDR adjustment).

Next, we investigated whether active transcriptional compensation takes place in tetraploid wheat. As each homoeolog group consists of only two homoeologs (rather than three), a loss‐of‐function mutation in one homoeolog would result in a 50% reduction in functional transcript, compared with 33% in hexaploid wheat. Thus, we hypothesised that active transcriptional compensation would be more likely to occur in tetraploid wheat. From six EMS‐mutagenised TILLING lines (cv Kronos; K2619, K2864, K3239, K0427, K4533, and K0774), a total of 101 unique homoeolog groups containing a PTC mutation were identified. In line with the results from hexaploid wheat, between 8.3% (in K0427) and 32.4% (in K2864) of the homoeologs with a PTC mutation were downregulated in the mutagenised lines compared with WT (FDR‐adjusted *P*‐value < 0.05; Fig. 2a). We found three homoeolog groups (3.0%) affected by a PTC mutation in which the nonmutated homoeolog was upregulated (FDR‐adjusted *P*‐value < 0.05; Fig. 2a; Table S1). The proportion of homoeolog groups with upregulation of the nonmutated homoeolog was not significantly different between those affected by a PTC mutation compared with those affected by a synonymous mutation (1.5% of homoeolog groups with a synonymous mutation, chi‐squared test *P*‐value = 0.234; Fig. 2b).

Despite the lack of widespread active transcriptional compensation between homoeologs in hexaploid and tetraploid wheat, we hypothesised that active transcriptional compensation may be responsible for the functional redundancy observed within particular homoeolog groups. The *PHS1* homoeolog group, encoding the plastidial α‐glucan phosphorylase, has been characterised as functionally redundant by Kamble *et al*. (2023) – the *phs1‐1* double mutant in tetraploid wheat has altered starch granule morphology (it has a decreased proportion of small starch granules and larger B‐type granules), while neither the *phs1‐A1* single mutant nor the *phs1‐B1* single mutant has altered granule morphology. By contrast, the *CSP41a* homoeolog group, encoding a chloroplast RNA‐binding protein, is nonredundant in tetraploid wheat – mutation of the A homoeolog alone (in the *csp41‐A* single mutant) is sufficient to increase resistance to yellow rust (Corredor‐Moreno *et al*., 2022). We used mutants in each of the homoeologs of *PHS1* and *CSP41a* to investigate whether the level of redundancy between homoeologs affected the degree of active transcriptional compensation observed (Table 2). For both homoeologs of *PHS1*, the presence of a PTC mutation was associated with downregulation of the affected homoeolog (for *PHS1‐A1*, PTC mutation in K4533, fold change = 0.18, FDR‐adjusted *P*‐value = 7.8 × 10^−19^; for *PHS1‐B1*, PTC mutation in K2864, fold change = 0.15, FDR‐adjusted *P*‐value = 4.3 × 10^−7^; Fig. 2c). However, there was no upregulation of the nonmutated homoeolog in either case (FDR‐adjusted *P*‐value > 0.05; Fig. 2c). Similarly, for *CSP41a*, the presence of a PTC mutation resulted in downregulation of the affected homoeolog (for *CSP41a‐A*, PTC mutation in K3239, fold change = 0.045, FDR‐adjusted *P*‐value = 1.9 × 10^−61^; for *CSP41a‐B*, PTC mutation in K2619, fold change = 0.093, FDR‐adjusted *P*‐value = 7.6 × 10^−15^; Fig. 2d). No upregulation of the nonmutated homoeolog was observed in either case (FDR‐adjusted *P*‐value > 0.05; Fig. 2d). This indicates that active transcriptional compensation is not necessary for functional redundancy between homoeologs.

## Discussion

We have shown, for 188 homoeolog groups in hexaploid across two independent experiments and 101 homoeolog groups in tetraploid wheat, that active transcriptional compensation occurs at a low frequency – only 2.5–3.3% of PTC mutations were associated with upregulation of the homoeologous genes across three independent datasets. These proportions were not significantly different from the proportion of synonymous mutations associated with homoeolog upregulation, suggesting that apparent compensatory upregulation of gene expression is not necessarily linked to the functional impact of the mutation (Figs 1, 2, S1).

Mechanisms to compensate for paralog loss‐of‐function have previously been shown to be nonuniversal – a similar study in yeast (*Saccharomyces cerevisiae*) found increased levels of *c*. 11% of proteins when their paralogs were deleted (DeLuna *et al*., 2010). DeLuna *et al*. (2010) also showed that compensatory upregulation occurred almost exclusively for proteins with essential functions, suggesting that upregulation is induced in response to a physiological deficit, rather than as a direct response to the mutation. Such ‘needs‐based’ responses could explain the upregulation observed in the homoeolog group encoding a subunit of Respiratory Complex I (Table S1; NADH dehydrogenase). However, this did not extend to any transcription factors, which are generally considered to be dosage‐sensitive (Birchler & Veitia, 2010), and might therefore be expected to be transcriptionally compensated to maintain gene–dosage balance.

The low level of active transcriptional compensation observed in wheat is not in keeping with the functional redundancy that is often observed between homoeologs. Although almost all of the mutagenised genes in our study are functionally uncharacterised, the *PHS1* homoeologs in tetraploid wheat, previously shown to be functionally redundant, also did not show active transcriptional compensation (Fig. 2c; Kamble *et al*., 2023). Future experiments to characterise the few genes that showed transcriptional compensation may provide additional insight into the connection to functional redundancy. It is possible that a different active compensatory mechanism facilitates functional redundancy between homoeologs – for example, Diss *et al*. (2014) propose translational upregulation or changes in protein localisation as modes of compensation. Our study is also unlikely to capture cell‐type or tissue‐specific transcriptional compensation, as highlighted by Iohannes & Jackson (2023). However, current models of transcriptional compensation in mouse, zebrafish and *C. elegans* indicate that expression of the PTC‐containing transcript can cell‐autonomously trigger compensation and paralog compensation is shared between different cell states, suggesting that tissue‐specificity may not be a major limitation (Sztal *et al*., 2018; Serobyan *et al*., 2020; Mellis *et al*., 2024). Alternatively, there may be significant passive compensation between homoeologs, with two‐thirds (in hexaploid wheat) or half (in tetraploid wheat) of the WT levels of functional transcript being sufficient to maintain the WT phenotype.

Passive compensation between homoeologs in wheat is probable given that these gene duplications are relatively young and have had less time to diverge in expression levels – the hybridisation events occurred between 500 000 (forming A and B subgenomes) and 10 000 yr ago (addition of the D subgenome; El Baidouri *et al*., 2017). Increased time since gene duplication is likely to result in hypofunctionalisation, as the paralogs acquire mutations potentially decreasing their expression, and compensatory drift, in which the expression of one paralog decreases over time and function is maintained by its highly expressed copy (Iohannes & Jackson, 2023). When hypofunctionalisation or compensatory drift have occurred, expression from one paralog is insufficient to maintain gene function and an active compensatory mechanism would be required for functional redundancy (Iohannes & Jackson, 2023). Accordingly, Cusack *et al*. (2021) found that the age of duplication is associated with functional redundancy in *A. thaliana*, with duplicates formed during the α‐WGD more likely to be functionally redundant than those that arose during the more ancient β‐ or γ‐WGD events. Furthermore, the *HBEGF* paralogs exhibiting active transcriptional compensation in zebrafish are much older than homoeologs in wheat, having arisen during the fish‐specific genome duplication event *c*. 320 million years ago (Ma; Vandepoele *et al*., 2004; Laisney *et al*., 2010; El‐Brolosy *et al*., 2019), and are thus more likely to have experienced hypofunctionalisation/compensatory drift. Similarly, the *CLV3* paralog in the Solanaceae, *CLE9*, arose at least 30 Ma and shows active transcriptional compensation (Rodriguez‐Leal *et al*., 2019). Overall, this underscores active transcriptional compensation as an evolutionary innovation that is only favourable when passive compensation is insufficient (whether to maintain functional redundancy or to adequately control gene expression noise, per Pires & Conant, 2016), and is otherwise unfavourable due to the cost of excess mRNA synthesis (with excess mRNA synthesis being selected against, as shown by Hausser *et al*., 2019).

Passive compensation between homoeologs suggests that targeting active transcriptional compensation mechanisms characterised in other species is unlikely to break functional redundancy in wheat and would not be a viable strategy for breeding. Rather, mechanisms to simultaneously introduce mutations in multiple/all homoeologs, such as gene editing, or to reduce the expression of multiple/all homoeologs, such as RNAi, are advantageous to tackle functional redundancy.

## Materials and Methods

### Plant materials

Target genes were selected based on the availability of multiple EMS‐mutagenised hexaploid wheat (*Triticum aestivum* L. cv *Cadenza*) lines (Krasileva *et al*., 2017) with PTC mutations either early or late within the coding sequence of the same gene. Further screening of publicly available gene expression data (Borrill *et al*., 2016; Ramírez‐González *et al*., 2018) was used to select genes with high expression in seedling leaf tissues. Based on these conditions, the genes *TraesCS5A02G119300* and *TraesCS6A02G155200* were chosen. Selected TILLING lines with PTC mutations in these genes are summarised in Table 1.

**Table 1:** Target genes and corresponding TILLING line mutations in Cadenza hexaploid wheat (*Triticum aestivum*).

Target gene	TILLING line	SNP	Amino acid change	Location within gene
*TraesCS5A02G119300*	Cadenza1015 (C1015)	C/T	W/*	Second exon (of 9)
	Cadenza1704 (C1704)	G/A	Q/*	Eighth exon (of 9)
*TraesCS6A02G155200*	Cadenza0895 (C0895)	G/A	W/*	First exon (of 2)
	Cadenza0604 (C0604)	G/A	W/*	Second exon (of 2)

SNP, single‐nucleotide polymorphism; TILLING, Targeting Induced Local Lesions in Genomes.

SSD lines were produced from the existing Cadenza TILLING M_5_ seed bulk for the selected lines (C0604, C0895, C1015, and C1704; Krasileva *et al*., 2017). For each line, a single M_6_ seed was sown and selfed in glasshouse conditions. The resulting M_7_ generation was grown in the field (Norwich, UK, 2017–2018) under standard agronomic practises in 1 m^2^ plots to bulk seeds. The M_8_ seeds were sown for the RNA‐sequencing experiment.

For tetraploid wheat, target genes were selected from previously characterised redundant and nonredundant genes. Targets were chosen based on the availability of EMS‐mutagenised tetraploid wheat (*T. turgidum* ssp. *durum* (Desf.) Husn. cv Kronos) with PTC mutations in the coding sequence (Krasileva *et al*., 2017) and high expression in seedling leaf tissues. Selected TILLING lines with PTC mutations in these genes are summarised in Table 2.

**Table 2:** Target genes and corresponding TILLING line mutations in Kronos tetraploid wheat (*Triticum turgidum* ssp. *durum)*.

Target gene	Homoeolog	TILLING line	SNP	Amino acid change	Location within gene
*PHS1*	*TraesCS5A02G395200*	Kronos4533 (K4533)	C/T	W/*	Second exon (of 15)
	*TraesCS5B02G400000*	Kronos2864 (K2864)	C/T	W/*	Fourth exon (of 15)
*CSP41a*	*TraesCS6A02G025700*	Kronos3239 (K3239)	G/A	Q/*	Third exon (of 6)
	*TraesCS6B02G036400*	Kronos2619 (K2619)	C/T	W/*	Fourth exon (of 6)

SNP, single‐nucleotide polymorphism; TILLING, Targeting Induced Local Lesions in Genomes.

In addition to Kronos4533, Kronos2864, Kronos3239, and Kronos2619, the TILLING lines Kronos0427 and Kronos0774 were grown to adjust for the lower mutation density in the Kronos TILLING lines. Seeds for the selected Kronos lines were obtained from the Germplasm Resources Unit, UK (www.seedstor.ac.uk).

### Plant growth conditions

Seeds were imbibed on damp filter paper at 4°C for 2 d and then moved to room temperature to promote germination for a further 2 d. Seeds were then sown into 96 cell trays containing John Innes F_2_ Starter + Grit (90% peat, 10% grit, 4 kg m^−3^ dolomitic limestone, 1.2 kg m^−3^ Osmocote Sart). Seedlings were grown in glasshouses at the John Innes Centre (Norwich, UK) with supplementary lighting and heating to maintain a minimum of 16 h light, with 18°C day and 15°C night. No obvious phenotypic differences or developmental abnormalities were observed in the mutant lines compared with WT at the seedling stage.

### DNA extraction and KASP genotyping

For Kronos, the TILLING lines were genotyped to ensure that they were homozygous for the PTC mutation of interest. DNA was extracted from seedling leaf tissue from 2‐wk‐old plants following the protocol from www.wheat‐training.com. The KASP primers used for genotyping are shown in Table S2. Genotyping was carried out using PACE mix (3CR Bioscience), according to the manufacturer's instructions. Genotype calls were made using the Kluster‐Caller software (v.3.4.1.36).

### RNA extraction and RT‐qPCR

Seedling leaf samples were taken from 2‐wk‐old plants (TILLING lines), frozen in liquid nitrogen, and stored at −70°C. RNA was extracted using the RNeasy Plant Mini Kit (Qiagen). Genomic DNA was digested using the RQ1 RNase‐free DNase (Promega). cDNA synthesis was carried out using M‐MLV reverse transcriptase (Invitrogen), according to the manufacturer's instructions. qPCR was carried out in a LightCycler 480 (Roche) using the LightCycler 480 SYBR Green I Master (Roche) with the following cycling conditions: initial start of 95°C for 5 min; 45 cycles of 10 s at 95°C, 15 s at 60°C, 30 s at 72°C, and reading for 1 s at 78°C. qPCR primers are shown in Table S2. Relative expression was determined by calculating the Pfaffl ratio for each transcript relative to the GAPDH transcript. The effect of genotype on relative expression was tested using a linear model and ANOVA in R (v.4.4.1).

### RNA extraction for RNA‐seq and analysis

Samples were taken from the second leaf of 2‐wk‐old seedlings, frozen in liquid nitrogen and stored at −70°C. For the Cadenza samples, RNA was extracted using the Spectrum Plant Total RNA Kit (Sigma) from three biological replicates for each TILLING line. For the Kronos samples, RNA was extracted using a TRIzol‐Chloroform method and cleaned with the RNA Clean & Concentrator Kit (Zymo Research, Irvine, CA, USA). Library preparation and sequencing were carried out by Novogene (Cambridge, UK), producing 150‐bp paired‐end reads. Reads were pseudoaligned to the IWGSC Chinese Spring v.1.1 transcriptome (IWGSC *et al*., 2018) and quantified using kallisto (v.0.46.1; Bray *et al*., 2016). For the Kronos samples, reads were pseudoaligned to the Chinese Spring transcriptome with transcripts from the D subgenome removed. An average of 28 429 382 reads per sample (81% of the total reads) were pseudoaligned for the Cadenza TILLING lines and an average of 27 411 192 reads per sample (74% of the total reads) were pseudoaligned for the Kronos TILLING lines. Abundance files from kallisto were imported into R (v.4.4.1) using Tximport (Soneson *et al*., 2015). Differentially expressed genes between the TILLING lines and WT were identified using DESeq2 (Love *et al*., 2014), applying a threshold of adjusted *P*‐value < 0.05. Upregulated genes were classified as those with fold change > 1, and downregulated genes as those with fold change < 1.

To identify variants present in the TILLING lines, the raw reads were trimmed with trimmomatic (v.0.39; parameters: ILLUMINACLIP:TruSeq3‐PE.fa:2:30:10 LEADING:3 TRAILING:3 SLIDINGWINDOW:4:15 MINLEN:80; Bolger *et al*., 2014) and mapped to the IWGSC Chinese Spring v.1.0 reference sequence (IWGSC *et al*., 2018) using Hisat2 (v.2.1.0; Pertea *et al*., 2016) and SAMtools (v.1.12; Danecek *et al*., 2021). Freebayes (v.1.2.0) was used for variant calling (parameters: ‐F 0.87 ‐‐min‐coverage 10 ‐‐use‐best‐n‐alleles 2; Garrison & Marth, 2012) and variant effect prediction was carried out using Vep (v.91.3; McLaren *et al*., 2016). Variants were filtered to keep only those predicted to cause a PTC or synonymous mutation and were homozygous. Further filtering was carried out to identify variants in homoeologous genes (using homoeolog groups defined in Evans *et al*., 2022), and those present in one of the TILLING lines, but not WT to account for cultivar specific single‐nucleotide polymorphism (SNPs). The lists of differentially expressed genes were then used to identify changes in expression in the genes affected by a PTC/synonymous mutation and their homoeologs. Homoeolog expression bias categories were calculated from transcripts per million for each of the homoeolog groups in the WT following the approach Euclidean distance approach described in Ramírez‐González *et al*. (2018). For the Kronos lines, the Euclidean distance approach was modified to account for the tetraploid genome architecture (with ideal expression ratios of 0.5 : 0.5 for balanced and 1 : 0 for dominant expression).

The same analysis was carried out on RNA‐sequencing data from H. C. Xiong *et al*. (2020), comparing two independent EMS‐mutagenised lines (*dm3* and *dm4*) to the Jing411 WT control.

## Competing interests

None declared.

## Author contributions

PB conceived the study. DD and PB designed the research and wrote the manuscript with input from EK and JS. JS generated the Cadenza SSD lines. DD and EK performed the experiments. DD carried out the analysis of RNA‐sequencing data and created the figures. All authors have read and approved the manuscript.

## Supporting information


**Fig. S1** Absence of widespread active transcriptional compensation in an independent RNA‐seq dataset of EMS‐mutagenised hexaploid wheat from H. C. Xiong *et al*. (2020).
**Fig. S2** PTC location does not affect whether transcriptional compensation occurs between homoeologs.
**Table S1** Homoeolog groups in which at least one nonmutated homoeolog is upregulated relative to the WT control.
**Table S2** Sequences of all primers used in this study.
Please note: Wiley is not responsible for the content or functionality of any Supporting Information supplied by the authors. Any queries (other than missing material) should be directed to the *New Phytologist* Central Office.

## Data Availability

The Cadenza SSD lines (Cadenza0604_SSD, Cadenza0895_SSD, and Cadenza1015_SSD) are available from the Germplasm Resources Unit (Norwich, United Kingdom; www.seedstor.ac.uk) through the Deposited Published Research Material Collection, with store codes DPRM0144, DPRM0145, DPRM0146, and DPRM0147 respectively. The Kronos EMS TILLING lines can be ordered from the Germplasm Resources Unit (Norwich, United Kingdom; www.seedstor.ac.uk) under accession nos.: Kronos0427, Kronos0774, Kronos2619, Kronos2864, Kronos3239, and Kronos4533. Raw reads from RNA‐sequencing have been deposited in the European Nucleotide Archive under project PRJEB89501. Scripts used for the analysis of the RNA‐sequencing data can be found on GitHub at www.github.com/Borrill‐Lab/Transcriptional_Compensation.

## References

[nph70646-bib-0001] Abe F , Haque E , Hisano H , Tanaka T , Kamiya Y , Mikami M , Kawaura K , Endo M , Onishi K , Hayashi T *et al*. 2019. Genome‐edited triple‐recessive mutation alters seed dormancy in wheat. Cell Reports 28: 1362–1369. 31365876 10.1016/j.celrep.2019.06.090

[nph70646-bib-0002] Bailon‐Zambrano R , Sucharov J , Mumme‐Monheit A , Murry M , Stenzel A , Pulvino AT , Mitchell JM , Colborn KL , Nichols JT . 2022. Variable paralog expression underlies phenotype variation. eLife 11: e79247. 36134886 10.7554/eLife.79247 PMC9555865

[nph70646-bib-0003] Birchler JA , Veitia RA . 2010. The gene balance hypothesis: implications for gene regulation, quantitative traits and evolution. New Phytologist 186: 54–62. 19925558 10.1111/j.1469-8137.2009.03087.x PMC2858765

[nph70646-bib-0004] Birchler JA , Yang H . 2022. The multiple fates of gene duplications: deletion, hypofunctionalization, subfunctionalization, neofunctionalization, dosage balance constraints, and neutral variation. Plant Cell 34: 2466–2474. 35253876 10.1093/plcell/koac076 PMC9252495

[nph70646-bib-0005] Bolger AM , Lohse M , Usadel B . 2014. Trimmomatic: a flexible trimmer for Illumina sequence data. Bioinformatics 30: 2114–2120. 24695404 10.1093/bioinformatics/btu170 PMC4103590

[nph70646-bib-0006] Borrill P , Adamski N , Uauy C . 2015. Genomics as the key to unlocking the polyploid potential of wheat. New Phytologist 208: 1008–1022. 26108556 10.1111/nph.13533

[nph70646-bib-0007] Borrill P , Ramirez‐Gonzalez R , Uauy C . 2016. expVIP: a customizable RNA‐seq data analysis and visualization platform. Plant Physiology 170: 2172–2186. 26869702 10.1104/pp.15.01667 PMC4825114

[nph70646-bib-0008] Bray NL , Pimentel H , Melsted P , Pachter L . 2016. Near‐optimal probabilistic RNA‐seq quantification. Nature Biotechnology 34: 525–527. 10.1038/nbt.3519 27043002

[nph70646-bib-0009] Corredor‐Moreno P , Badgami R , Jones S , Saunders DGO . 2022. Temporally coordinated expression of nuclear genes encoding chloroplast proteins in wheat promotes *Puccinia striiformis* f. sp. *tritici* infection. Communications Biology 5: 853. 35996019 10.1038/s42003-022-03780-4 PMC9395331

[nph70646-bib-0010] Cusack SA , Wang PP , Lotreck SG , Moore BM , Meng FR , Conner JK , Krysan PJ , Lehti‐Shiu MD , Shiu SH . 2021. Predictive models of genetic redundancy in *Arabidopsis thaliana* . Molecular Biology and Evolution 38: 3397–3414. 33871641 10.1093/molbev/msab111 PMC8321531

[nph70646-bib-0011] Danecek P , Bonfield JK , Liddle J , Marshall J , Ohan V , Pollard MO , Whitwham A , Keane T , McCarthy SA , Davies RM *et al*. 2021. Twelve years of SAMtools and BCFtools . GigaScience 10: giab008. 33590861 10.1093/gigascience/giab008 PMC7931819

[nph70646-bib-0012] Degenhardt RF , Bonham‐Smith PC . 2008. Transcript profiling demonstrates absence of dosage compensation in *Arabidopsis* following loss of a single *RPL23a* paralog. Planta 228: 627–640. 18566829 10.1007/s00425-008-0765-6

[nph70646-bib-0013] DeLuna A , Springer M , Kirschner MW , Kishony R . 2010. Need‐based up‐regulation of protein levels in response to deletion of their duplicate genes. PLoS Biology 8: e1000347. 20361019 10.1371/journal.pbio.1000347 PMC2846854

[nph70646-bib-0014] Diss G , Ascencio D , DeLuna A , Landry CR . 2014. Molecular mechanisms of paralogous compensation and the robustness of cellular networks. Journal of Experimental Zoology Part B‐Molecular and Developmental Evolution 322: 488–499. 24376223 10.1002/jez.b.22555

[nph70646-bib-0015] El Baidouri M , Murat F , Veyssiere M , Molinier M , Flores R , Burlot L , Alaux M , Quesneville H , Pont C , Salse J . 2017. Reconciling the evolutionary origin of bread wheat (*Triticum aestivum*). New Phytologist 213: 1477–1486. 27551821 10.1111/nph.14113

[nph70646-bib-0016] El‐Brolosy MA , Kontarakis Z , Rossi A , Kuenne C , Günther S , Fukuda N , Kikhi K , Boezio GLM , Takacs CM , Lai SL *et al*. 2019. Genetic compensation triggered by mutant mRNA degradation. Nature 568: 193–197. 30944477 10.1038/s41586-019-1064-z PMC6707827

[nph70646-bib-0017] Evans CEB , Arunkumar R , Borrill P . 2022. Transcription factor retention through multiple polyploidization steps in wheat. G3: Genes, Genomes, Genetics 12: jkac147. 35748743 10.1093/g3journal/jkac147 PMC9339333

[nph70646-bib-0018] Garrison E , Marth G . 2012. Haplotype‐based variant detection from short‐read sequencing. doi: 10.48550/arXiv.1207.3907.

[nph70646-bib-0019] Hausser J , Mayo A , Keren L , Alon U . 2019. Central dogma rates and the trade‐off between precision and economy in gene expression. Nature Communications 10: 68. 10.1038/s41467-018-07391-8 PMC6325141 30622246

[nph70646-bib-0020] Iohannes SD , Jackson D . 2023. Tackling redundancy: genetic mechanisms underlying paralog compensation in plants. New Phytologist 240: 1381–1389. 37724752 10.1111/nph.19267

[nph70646-bib-0021] IWGSC , Appels R , Eversole K , Stein N , Feuillet C , Keller B , Rogers J , Pozniak CJ , Choulet F , Distelfeld A *et al*. 2018. Shifting the limits in wheat research and breeding using a fully annotated reference genome. Science 361: eaar7191. 30115783 10.1126/science.aar7191

[nph70646-bib-0022] Kamble NU , Makhamadjonov F , Fahy B , Martins C , Saalbach G , Seung D . 2023. Initiation of B‐type starch granules in wheat endosperm requires the plastidial ɑ‐glucan phosphorylase PHS1. Plant Cell 35: 4091–4110. 37595145 10.1093/plcell/koad217 PMC10615211

[nph70646-bib-0023] Krasileva KV , Vasquez‐Gross HA , Howell T , Bailey P , Paraiso F , Clissold L , Simmonds J , Ramirez‐Gonzalez RH , Wang XD , Borrill P *et al*. 2017. Uncovering hidden variation in polyploid wheat. Proceedings of the National Academy of Sciences, USA 114: E913–E921. 10.1073/pnas.1619268114 PMC5307431 28096351

[nph70646-bib-0024] Kwon CT , Tang LL , Wang XG , Gentile I , Hendelman A , Robitaille G , Van Eck J , Xu C , Lippman ZB . 2022. Dynamic evolution of small signalling peptide compensation in plant stem cell control. Nature Plants 8: 346–355. 35347264 10.1038/s41477-022-01118-w

[nph70646-bib-0025] Laisney JAGC , Braasch I , Walter RB , Meierjohann S , Schartl M . 2010. Lineage‐specific co‐evolution of the Egf receptor/ligand signaling system. BMC Evolutionary Biology 10: 27. 20105326 10.1186/1471-2148-10-27 PMC2834686

[nph70646-bib-0045] Love MI , Huber W , Anders S . 2014. Moderated estimation of fold change and dispersion for RNA-seq data with DESeq2. Genome Biology 15: 550. 25516281 10.1186/s13059-014-0550-8 PMC4302049

[nph70646-bib-0026] Ma ZP , Zhu PP , Shi H , Guo LW , Zhang QH , Chen YN , Chen SM , Zhang Z , Peng JR , Chen J . 2019. PTC‐bearing mRNA elicits a genetic compensation response via Upf3a and COMPASS components. Nature 568: 259–263. 30944473 10.1038/s41586-019-1057-y

[nph70646-bib-0027] Matsuoka Y . 2011. Evolution of polyploid *Triticum* wheats under cultivation: the role of domestication, natural hybridization and allopolyploid speciation in their diversification. Plant and Cell Physiology 52: 750–764. 21317146 10.1093/pcp/pcr018

[nph70646-bib-0028] McLaren W , Gil L , Hunt SE , Riat HS , Ritchie GR , Thormann A , Flicek P , Cunningham F . 2016. The ensembl variant effect predictor. Genome Biology 17: 122. 27268795 10.1186/s13059-016-0974-4 PMC4893825

[nph70646-bib-0029] Mellis IA , Melzer ME , Bodkin N , Goyal Y . 2024. Prevalence of and gene regulatory constraints on transcriptional adaptation in single cells. Genome Biology 25: 217. 39135102 10.1186/s13059-024-03351-2 PMC11320884

[nph70646-bib-0030] Panchy N , Lehti‐Shiu M , Shiu SH . 2016. Evolution of gene duplication in plants. Plant Physiology 171: 2294–2316. 27288366 10.1104/pp.16.00523 PMC4972278

[nph70646-bib-0031] Pertea M , Kim D , Pertea GM , Leek JT , Salzberg SL . 2016. Transcript‐level expression analysis of RNA‐seq experiments with HISAT, StringTie and Ballgown. Nature Protocols 11: 1650–1667. 27560171 10.1038/nprot.2016.095 PMC5032908

[nph70646-bib-0032] Pires JC , Conant GC . 2016. Robust yet fragile: expression noise, protein misfolding, and gene dosage in the evolution of genomes. Annual Review of Genetics 50: 113–131. 10.1146/annurev-genet-120215-035400 27617972

[nph70646-bib-0033] Ramírez‐González RH , Borrill P , Lang D , Harrington SA , Brinton J , Venturini L , Davey M , Jacobs J , van Ex F , Pasha A *et al*. 2018. The transcriptional landscape of polyploid wheat. Science 361: eaar6089. 30115782 10.1126/science.aar6089

[nph70646-bib-0034] Rodriguez‐Leal D , Xu C , Kwon CT , Soyars C , Demesa‐Arevalo E , Man J , Liu L , Lemmon ZH , Jones DS , Van Eck J *et al*. 2019. Evolution of buffering in a genetic circuit controlling plant stem cell proliferation. Nature Genetics 51: 786–792. 30988512 10.1038/s41588-019-0389-8 PMC7274162

[nph70646-bib-0035] Schreiber AW , Hayden MJ , Forrest KL , Kong SL , Langridge P , Baumann U . 2012. Transcriptome‐scale homoeolog‐specific transcript assemblies of bread wheat. BMC Genomics 13: 492. 22989011 10.1186/1471-2164-13-492 PMC3505470

[nph70646-bib-0036] Serobyan V , Kontarakis Z , El‐Brolosy MA , Welker JM , Tolstenkov O , Saadeldein AM , Retzer N , Gottschalk A , Wehman AM , Stainier DYR . 2020. Transcriptional adaptation in *Caenorhabditis elegans* . eLife 9: e50014. 31951195 10.7554/eLife.50014 PMC6968918

[nph70646-bib-0037] Singh M , Kumar M , Thilges K , Cho MJ , Cigan AM . 2017. MS26/CYP704B is required for anther and pollen wall development in bread wheat (*Triticum aestivum* L.) and combining mutations in all three homeologs causes male sterility. PLoS ONE 12: e0177632. 28520767 10.1371/journal.pone.0177632 PMC5433722

[nph70646-bib-0038] Soneson C , Love MI , Robinson MD . 2015. Differential analyses for RNA‐seq: transcript‐level estimates improve gene‐level inferences. F1000Research 4: 1521. 26925227 10.12688/f1000research.7563.1 PMC4712774

[nph70646-bib-0039] Sztal TE , McKaige EA , Williams C , Ruparelia AA , Bryson‐Richardson RJ . 2018. Genetic compensation triggered by actin mutation prevents the muscle damage caused by loss of actin protein. PLoS Genetics 14: e1007212. 29420541 10.1371/journal.pgen.1007212 PMC5821405

[nph70646-bib-0040] Sztal TE , Stainier DYR . 2020. Transcriptional adaptation: a mechanism underlying genetic robustness. Development 147: dev186452. 32816903 10.1242/dev.186452

[nph70646-bib-0041] Uauy C , Wulff BBH , Dubcovsky J . 2017. Combining traditional mutagenesis with new high‐throughput sequencing and genome editing to reveal hidden variation in polyploid wheat. Annual Review of Genetics 51: 435–454. 10.1146/annurev-genet-120116-024533 28934591

[nph70646-bib-0042] Vandepoele K , De Vos W , Taylor JS , Meyer A , Van de Peer Y . 2004. Major events in the genome evolution of vertebrates: paranome age and size differ considerably between ray‐finned fishes and land vertebrates. Proceedings of the National Academy of Sciences, USA 101: 1638–1643. 10.1073/pnas.0307968100 PMC341801 14757817

[nph70646-bib-0043] Xiong HC , Zhou CY , Guo HJ , Xie YD , Zhao LS , Gu JY , Zhao SR , Ding YP , Liu LX . 2020. Transcriptome sequencing reveals hotspot mutation regions and dwarfing mechanisms in wheat mutants induced by γ‐ray irradiation and EMS. Journal of Radiation Research 61: 44–57. 31825082 10.1093/jrr/rrz075 PMC6976738

[nph70646-bib-0044] Xiong W , Chen XZ , Zhu CX , Zhang JC , Lan T , Liu L , Mo BX , Chen XM . 2020. Arabidopsis paralogous genes *RPL23aA* and *RPL23aB* encode functionally equivalent proteins. BMC Plant Biology 20: 463. 33032526 10.1186/s12870-020-02672-1 PMC7545930

