# Supplementary material for: Homoeolog expression in polyploid wheat mutants shows limited transcriptional compensation
Source: New Phytol. 2025 Oct 13;249(1):16–23. doi: 10.1111/nph.70646 (PMC12676073; doi:10.1111/nph.70646)
Supplement: Supplementary file 1 — Fig. S1 Absence of widespread active transcriptional compensation in an independent RNA‐seq dataset of EMS‐mutagenised hexaploid wheat from H. C. Xiong et al. (2020). Fig. S2 PTC location does not affect whether transcriptional compensation occurs between homoeologs. Table S1 Homoeolog groups in which at least one nonmutated homoeolog is upregulated relative to the WT control. Table S2 Sequences of all primers used in this study. Please note: Wiley is not responsible for the content or functionality of any Supporting Information supplied by the authors. Any queries (other than missing material) should be directed to the New Phytologist Central Office. [file NPH-249-16-s001.pdf]

New Phytologist Supporting Information

Article title: Homoeolog expression in polyploid wheat mutants shows limited transcriptional compensation

Authors: Delfi Dorussen[1], Emilie Knight[1], James Simmonds[1], Philippa Borrill[1*]

[1] Department of Crop Genetics, John Innes Centre, Norwich Research Park, Norwich, NR4 7UH, United Kingdom

* Corresponding author, email: philippa.borrill@jic.ac.uk

Article acceptance date: 18 September 2025

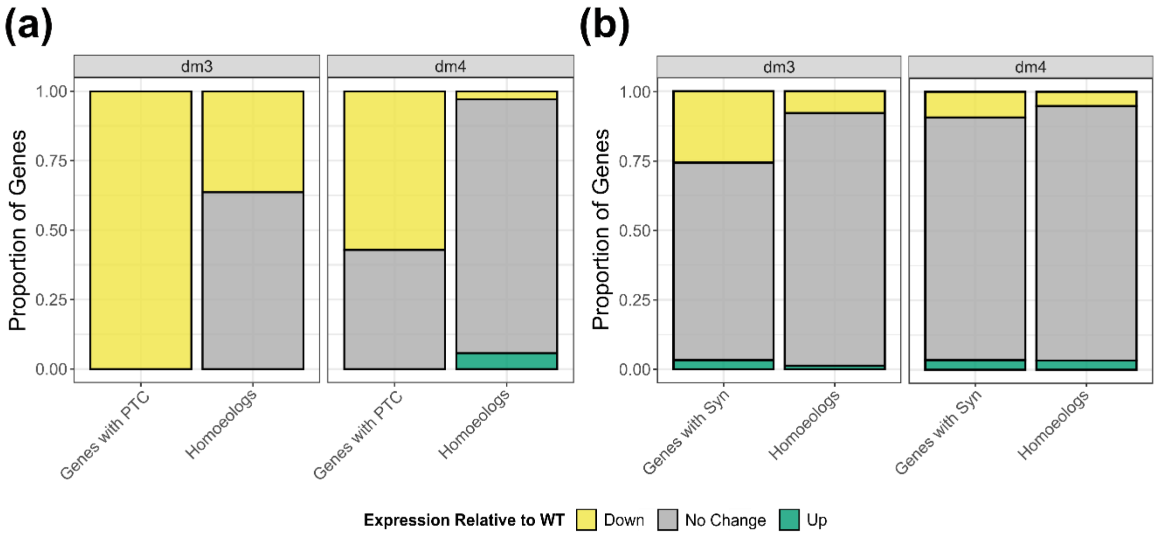

Fig. S1. Absence of widespread active transcriptional compensation in an independent RNA-seq dataset of EMS-mutagenised hexaploid wheat from Xiong, HC *et al.* (2020). (a) Expression of genes with a premature termination codon (PTC) mutation (left bar) and their homoeologs (right bar) in each of the EMS-mutagenised lines (*dm3* and *dm4*). (b) Expression of genes with a synonymous mutation (left bar) and their homoeologs (right bar) in each of the EMS-mutagenised lines (dm3 and dm4). In (a) and (b) the proportion of genes that are down-regulated (FDR adjusted p-value < 0.05) is shown in yellow, up-regulated (FDR adjusted p-value < 0.05) in green, and no change in expression in grey, all relative to Jing411 WT.

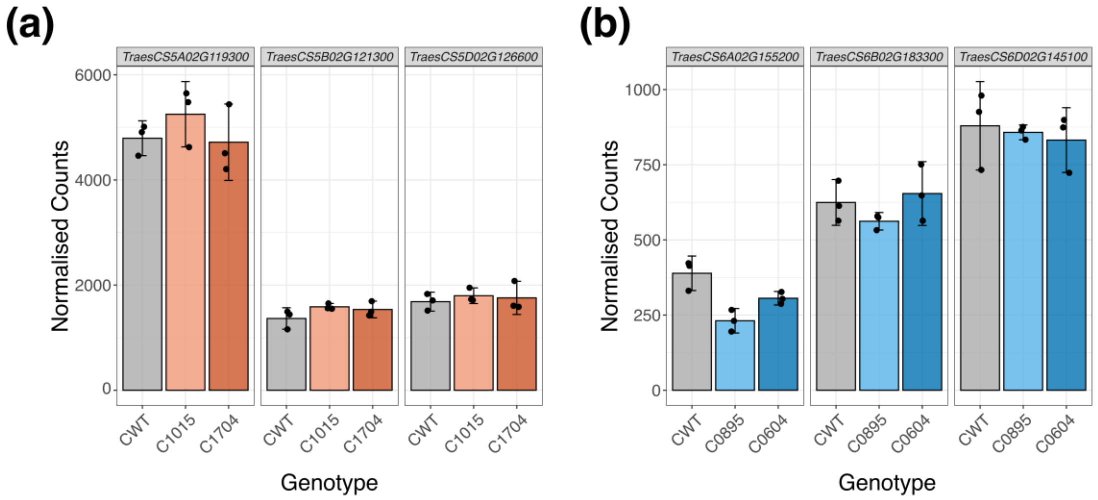

Fig. S2. PTC location does not affect whether active transcriptional compensation occurs between homoeologs. (a) The DESeq2 normalised counts for *TraesCS5A02G119300* and its homoeologs (*TraesCS5B02G121300* and *TraesCS5D02G126600*) in Cadenza WT (CWT) (grey), C1015 (light orange), and C1704 (dark orange). (b) The DESeq2 normalised counts for *TraesCS6A02G155200* and its homoeologs (*TraesCS6B02G183300* and *TraesCS6D02G145100*) in Cadenza WT (CWT) (grey), C0895 (light blue), and C0604 (dark blue). Error bars represent the 95% confidence interval around the mean, estimated as 1.96 x standard error.

Supplementary Tables

Table S1. Homoeolog groups in which at least one non-mutated homoeolog is up-regulated relative to WT control.

Mutant Line/ Source	Annotation of Homoeolog Group	Homoeologs	PTC Mutation	Fold Change	DESeq2 Adjusted p-value	Expression Relative to WT	Expression Category in WT (ratio A, B, D expression; A, B for tetraploid K0774)
C0604	MFS transporter (IPR036259); proton-dependent oligopeptide transporter (IPR000109)	*TraesCS1A02G269600*	No	1.98	0.000317	Up	Balanced (0.33, 0.30, 0.37)
		TraesCS1B02G280100	No	1.47	0.134	-	
		TraesCS1D02G269700	Yes (Exon 4/4; AA 529/576)	1.74	0.0201	Up	
C0895	Meiosis regulator and mRNA stability factor 1 (IPR024768)	*TraesCS2A02G150200*	No	3.48	0.0125	Up	Balanced (0.27, 0.39, 0.34)
		TraesCS2B02G175400	Yes (Exon 2/2; AA 152/632)	0.50	0.540	-	
		TraesCS2D02G155300	No	0.64	0.559	-	
C1015	MFS transporter (IPR036259); sugar phosphate transporter (IPR000849)	*TraesCS7A02G300800*	No	1.76	0.0349	Up	D-suppressed (0.33, 0.52, 0.14)
		TraesCS7B02G201500	No	1.53	0.0609	-	
		TraesCS7D02G296500	Yes (Exon 1/2; AA 272/559)	1.08	0.912	-	
	Serine/ threonine protein kinase (IPR011009; IPR008271)	*TraesCS7A02G544000*	Yes (Exon 1/1; AA 26/413)	2.16	0.342	-	B-dominant (0.12, 0.69, 0.18)
		TraesCS7B02G466300	No	1.03	0.970	-	
		TraesCS7D02G530500	No	4.31	0.000108	Up	
dm4 (Xiong HC *et al.*, 2020)	NADH dehydrogenase [ubiquinone] (complex I), alpha subcomplex subunit 1 (IPR017384)	*TraesCS1A02G302900*	Yes (Exon 1/3; AA 8/66)	0.11	7.31×10^{-82}	Down	Balanced (0.31, 0.37, 0.31)
		TraesCS1B02G313200	No	1.45	0.0130	Up	
		TraesCS1D02G302300	No	1.45	0.00348	Up	

K0774	Cytokinin dehydrogenase (IPR016170; IPR015345)	*TraesCS1A02G159600*	No	2.65	0.00183	Up	Balanced<br><br>(0.57, 0.43)
		TraesCS1B02G176000	Yes (Exon 5/6; AA 338/522)	2.07	0.0329	Up	
	ATP-dependent Clp protease/ Chaperone ClpA/ClpB (IPR050130)	*TraesCS2A02G291100*	Yes (Exon 5/12; AA 420/946)	1.59	0.162	-	Balanced<br><br>(0.28, 0.72)
		TraesCS2B02G307500	No	1.76	0.00771	Up	
	Squalene/ phytoene synthase (IPR002060)	*TraesCS5A02G020900*	Yes (Exon 1/6; AA 27/396)	0.63	0.189	-	A-dominant<br><br>(0.81, 0.19)
		TraesCS5B02G017900	No	2.64	0.00860	Up	

Table S2. Sequences of all primers used in this study. For KASP genotyping, the VIC tail (GAAGGTCGGAGTCAACGGATT) was added to the 5' end of the WT allele primers, and the FAM tail (GAAGGTGACCAAGTTCATGCT) was added to the 5' end of the mutant allele primers.

Name of Primer	Sequence (5'-3')	Purpose
Gene4_F1_Aspe	CTACCGATTCTTGGAGGAAGG	qPCR; forwards primer for A homoeolog of *TraesCS5A02G119300*
Gene4_F2_Bspe	ATGGAGCTTCTGTCTCACATGT	qPCR; forwards primer for B homoeolog of *TraesCS5A02G119300*
Gene4_F1_Dspe	CTACCGATTCTTGGAGGAACA	qPCR; forwards primer for D homoeolog of *TraesCS5A02G119300*
Gene4_R1	CCCATTCAGGTTCTGCTTTCT	qPCR; reverse primer for A and D homoeologs of *TraesCS5A02G119300*
Gene4_R2	TGATTCTGGATGTGCTTCCT	qPCR; reverse primer for B homoeolog of *TraesCS5A02G119300*
Gene3_F1_Aspe	GCTGCTGGAGAAGCCTGAA	qPCR; forwards primer for A homoeolog of *TraesCS6A02G155200*
Gene3_F1_Bspe	GCTGCTGGAGAAGCCTGAG	qPCR; forwards primer for B homoeolog of *TraesCS6A02G155200*
Gene3_F1_Dspe	GCTGCTGGAGAAGCCTGAC	qPCR; forwards primer for D homoeolog of *TraesCS6A02G155200*
Gene3_R1	ATATTTGGGTGCCTTAGGGTAGA	qPCR; reverse primer for A, B and D homoeologs of *TraesCS6A02G155200*
TaGAPDH_F	TTAGACTTGCGAAGCCAGCA	qPCR; forwards primer for GAPDH control
TaGAPDH_R	AAATGCCCTTGAGGTTTCCC	qPCR; reverse primer for GAPDH control
PHS1-A_K4533_WT	TTTTTGATTCTCTGATATTGAATTG	KASP genotyping; WT primer for K4533 (*phs1-a1* mutant); from Kamble *et al*. (2023)
PHS1-A_K4533_Mut	TTTTTGATTCTCTGATATTGAATTA	KASP genotyping; mutant primer for K4533 (*phs1-a1* mutant); from Kamble *et al*. (2023)
PHS1-A_K4533_Com	AAGGTATGAGATAAGGCTGAAGA	KASP genotyping; common primer for K4533 (*phs1-a1* mutant); from Kamble *et al*. (2023)
PHS1-B_K2864_WT	AGAGACATCATTTCTTACGATCTCC	KASP genotyping; WT primer for K2864 (*phs1-b1* mutant); from Kamble *et al*. (2023)
PHS1-B_K2864_Mut	AGAGACATCATTTCTTACGATCTCT	KASP genotyping; mutant primer for K2864 (*phs1-b1* mutant); from Kamble *et al*. (2023)

PHS1-B_K2864_Com	ACCTGCATGTTAGCTTCTTTTCT	KASP genotyping; common primer for K2864 (*phs1-b1* mutant); from Kamble *et al*. (2023)
CSP41a-A_K3239_WT	GCTGCTGATGAACAGGAACTG	KASP genotyping; WT primer for K3239 (*csp41-a* mutant); from Corredor-Moreno *et al*. (2022)
CSP41a-A_K3239_Mut	GCTGCTGATGAACAGGAACTA	KASP genotyping; mutant primer for K3239 (*csp41-a* mutant); from Corredor-Moreno *et al*. (2022)
CSP41a-A_K3239_Com	AGTTTTGATGTTTGGTTGCGT	KASP genotyping; common primer for K3239 (*csp41-a* mutant); from Corredor-Moreno *et al*. (2022)
CSP41a-B_K2619_WT_1	GCGGAGTTCGGCAGCTGG	KASP genotyping; WT primer for K2619 (*csp41-b* mutant)
CSP41a-B_K2619_Mut_1	GCGGAGTTCGGCAGCTGA	KASP genotyping; mutant primer for K2619 (*csp41-b* mutant)
CSP41a-B_K2619_Com_1a	CTCGCAGTCCTTGTTGTTGC	KASP genotyping; common primer for K2619 (*csp41-b* mutant)
